# Binding Potassium to Improve Treatment With Renin-Angiotensin-Aldosterone System Inhibitors: Results From Multiple One-Stage Pairwise and Network Meta-Analyses of Clinical Trials

**DOI:** 10.3389/fmed.2021.686729

**Published:** 2021-08-19

**Authors:** Frank Lizaraso-Soto, Eduardo Gutiérrez-Abejón, Juan Bustamante-Munguira, Débora Martín-García, María Montserrat Chimeno, Álvaro Nava-Rebollo, Álvaro Maurtua-Briseño-Meiggs, Darío Fernández-Zoppino, Elena Bustamante-Munguira, Félix Jesús de Paz, Jesús Grande-Villoria, Carlos Ochoa-Sangrador, Manuel Pascual, F. Javier Álvarez, Francisco Herrera-Gómez

**Affiliations:** ^1^Pharmacological Big Data Laboratory, University of Valladolid, Valladolid, Spain; ^2^Centro de Investigación en Salud Pública, Instituto de Investigación de la Facultad de Medicina Humana, Universidad de San Martín de Porres, Lima, Peru; ^3^Technical Direction of Pharmaceutical Assistance, Gerencia Regional de Salud de Castilla y León, Valladolid, Spain; ^4^Cardiac Surgery Department, University Clinical Hospital of Valladolid, Valladolid, Spain; ^5^Clinical Nephrology Unit, University Clinical Hospital of Valladolid, Valladolid, Spain; ^6^Internal Medicine Department, Hospital Virgen de la Concha, Zamora, Spain; ^7^Nephrology Department, Hospital Virgen de la Concha, Zamora, Spain; ^8^Woodland Medical Practice—NHS, Lincolnshire, United Kingdom; ^9^Department of Health Sciences, Faculty of Health Science, University of Burgos, Burgos, Spain; ^10^Intensive Care Unit, University Clinical Hospital of Valladolid, Valladolid, Spain; ^11^Clinical Epidemiology Support Office, Sanidad de Castilla y León, Zamora, Spain; ^12^Transplantation Center, Lausanne University Hospital and University of Lausanne, Lausanne, Switzerland; ^13^Ethics Committee of Drug Research–East Valladolid Area, University Clinical Hospital of Valladolid, Valladolid, Spain; ^14^Castile and León's Research Consolidated Unit n° 299, Valladolid, Spain

**Keywords:** hyperkalemia, mineralocorticoid receptor antagonists, nanomedicine, meta-analysis (as topic), potassium-binding polymers

## Abstract

This manuscript presents findings from the first dichotomous data pooling analysis on clinical trials (CT) regarding the effectiveness of binding potassium. The results emanated from pairwise and network meta-analyses aiming evaluation of response to commercial potassium-binding polymers, that is, to achieve and maintain normal serum potassium (*n* = 1,722), and the association between this response and an optimal dosing of renin-angiotensin-aldosterone system inhibitors (RAASi) needing individuals affected by heart failure (HF) or resistant hypertension, who may be consuming other hyperkalemia-inducing drugs (HKID) (e.g., β-blockers, heparin, etc.), and frequently are affected by chronic kidney disease (CKD) (*n* = 1,044): According to the surface under the cumulative ranking area (SUCRA), sodium zirconium cyclosilicate (SZC) (SUCRA >0.78), patiromer (SUCRA >0.58) and sodium polystyrene sulfonate (SPS) (SUCRA <0.39) were different concerning their capacity to achieve normokalemia (serum potassium level (sK+) 3.5–5.0 mEq/L) or acceptable kalemia (sK+ ≤ 5.1 mEq/L) in individuals with hyperkalemia (sK+ >5.1 mEq/L), and, when normokalemia is achieved, patiromer 16.8–25.2 g/day (SUCRA = 0.94) and patiromer 8.4–16.8 g/day (SUCRA = 0.41) can allow to increase the dose of spironolactone up to 50 mg/day in subjects affected by heart failure (HF) or with resistant hypertension needing treatment with other RAASi. The potential of zirconium cyclosilicate should be explored further, as no data exists to assess properly its capacity to optimize dosing of RAASi, contrarily as it occurs with patiromer. More research is also necessary to discern between benefits of binding potassium among all type of hyperkalemic patients, for example, patients with DM who may need treatment for proteinuria, patients with early hypertension, etc.

**Systematic Review Registration:**https://www.crd.york.ac.uk/PROSPERO/, identifier: CRD42020185614, CRD42020185558, CRD42020191430.

## Introduction

Hyperkalemia [serum potassium level (sK+): >5.1 mEq/L] is a life-threatening situation. Individuals with heart failure (HF) may be affected, particularly when they present chronic kidney disease (CKD). Indeed, potassium excretion is impaired in a non-acute manner in such individuals, and physicians are aware of avoiding this situation when estimated glomerular filtration rate (eGFR) falls to 60 ml/min/1.73 m^2^, that is, in patients into the Kidney Disease–Improving Global Outcomes (KDIGO) GFR categories G3a–G5 (1). HF patients need treatment with angiotensin-converting enzyme inhibitors (ACEi) and/or angiotensin II receptor blockers (ARB), and, in most cases, the addition of a mineralocorticoid receptor antagonist (MRA) is imperative (2).

Treatment with MRA in addition to other renin-angiotensin-aldosterone system inhibitors (RAASi) is also frequently observed among patients with resistant hypertension (i.e., uncontrolled blood pressure when tacking three or more classes of antihypertensive drugs, one of which is a diuretic). Resistant hypertension patients should not be considered as patients with hypertension responding to drugs. These individuals are also affected frequently by CKD, and a sizable proportion of them present some degree of HF (3).

Hyperkalemia is also a concern among individuals with diabetes mellitus (DM), especially with uncontrolled glycemia levels, and for those taking RAASi (e.g., from early hypertension stages, for controlling diabetic proteinuria, etc.), especially when needing other hyperkalemia-inducing drugs (HKID) (e.g., β-blockers, heparin, etc.).

### Hypothesis and Study Objective

RAASi have demonstrated to improve mortality and other patient-relevant outcomes in HF and resistant hypertension, and substantial benefits in other conditions (e.g., from early hypertension stages patients, to treat diabetic proteinuria, etc.); however, hyperkalemia limits the use of optimal doses of these drugs (4). In figures, hyperkalemia affects globally approximately 23 millions of people having HF and 100 millions of people affected by resistant hypertension (5, 6). In this sense, potassium-binding polymers may allow an optimal treatment with RAASi (7); particularly, new polymers patiromer [Anatomical Therapeutic Chemical (ATC) code: V03AE09] and sodium zirconium cyclosilicate (SZC) (ATC code: V03AE10) face their attractive safety profile compared to classic molecules (8).

Notwithstanding, in order to elucidate the beneficial impact of binding potassium, Bayesian network meta-analysis are necessary to evaluate the capacity of such polymers to facilitate optimal dosing of RAASi, after confirmation of independent effects from all available potassium-binding polymers on correction of hyperkalemia (i.e., efficacy as treatment agent) and on maintaining normal serum potassium (i.e., efficacy as preventive agent) (9).

Individuals along the wide spectrum of CKD should be considered into the evaluation of these nanodrugs, that is, not only patients into KDIGO GFR categories G3a to G5, but also patients with early CKD (KDIGO GFR categories G1 and G2) including subjects with normal kidney function (NKF), as well as dialysis patients and kidney transplant recipients (KTR), as performed in other pharmacometrical studies assessing drugs to treat CKD patients (10).

This manuscript presents findings from pairwise and network meta-analyses aiming evaluation of the efficacy of commercial potassium-binding polymers (i.e., their capacity to achieve and maintain normal serum potassium), and the association between this efficacy and optimal dosing in RAASi-based schemes needing individuals affected by HF or resistant hypertension.

## Materials and Methods

A dichotomous effects meta-analysis following a multiple parallel one-stage systematic review design was performed on clinical trials (CT) having assessed the use of commercial potassium-binding polymers to treat and prevent hyperkalemia. The analysis presented here was not intended to present a summary of continuous data on binding potassium, nor a qualitative systematic review of evidence on these drugs.

Independent study searching, screening, selection, data extraction, and synthesis were carried out in accordance with the Preferred Reporting Items for Systematic reviews and Meta-Analyses (PRISMA) recommendations (11), and its extension statement for the reporting of systematic reviews incorporating network meta-analyses of healthcare interventions (12). Such details and those on registration and prospective study updating are consultable online at the site of the International Prospective Register of Systematic Reviews PROSPERO on https://www.crd.york.ac.uk/PROSPERO/ (reference IDs: CRD42020185614, CRD42020185558, CRD42020191430).

### Systematic Review Question Elements, Literature Search, and Synthesis Strategy

Study participants were subjects affected by or at risk of developing hyperkalemia, and they may have HF or resistant hypertension, and may have NKF, CKD into KDIGO GFR categories G1–G5, end-stage kidney disease (ESKD) necessitating dialysis, or received a kidney transplant.

The main intervention was binding potassium either used with the intention to treat or prevent hyperkalemia. Classical and new commercial potassium-binding polymers were considered. Treatment with RAASi, HKID (e.g., β-blockers, heparin, etc.), dietary restriction, diuretics, insulin and other antidiabetic drugs, phosphate-binding drugs, were considered as co-interventions. Comparators were, for randomized controlled trials, placebo and potassium-binding polymers at lower doses, and, for single-arm trials, pre-treatment state.

The same syntaxis of search formulae was used in the three systematic reviews performed. Search formulae was constructed by associating the name of each potassium-binding polymer, including trade names, ATC code, and other names, and the main indication of these drugs, that is, hyperkalemia, considering all possible term variations (e.g., potassium, hyperpotassemia, and hyperkalemia). Searches were not filtered by report type, access type (e.g., open access), or publication year/date. Published studies were searched in MEDLINE (PubMed, Ovid and Web of Science), EMBASE (Elsevier's Scopus), and in Cochrane Controlled Register of Trials (CENTRAL), up to June 2020. Study registries (ClinicalTrials.gov, the EU Clinical Trials Register, and the United Kingdoms' ISRCTN registry) and unpublished study sources (PhD and Master theses, meeting abstracts archives) were also searched. The reference lists of included studies were scanned to identify all relevant studies cited by included studies, so that they did not remain unnoticed.

Risk of bias assessment using the standard tool produced by the Cochrane Collaboration (13) preceded data synthesis.

This meta-analysis presents effect estimates on aggregate data. The first-step analysis assessed the efficacy of binding potassium (i.e., the capacity of commercial potassium-binding polymers to achieve and maintain normal serum potassium) at network and pairwise level. Pooled odds ratios (OR) and 95% credible intervals (95% CrI) for the outcomes of normokalemia (sK+ 3.5 to 5.0 mEq/L) and acceptable kalemia (sK+ ≤ 5.1 mEq/L) corresponding to each of the assessed potassium-binding polymers at all possible doses, were obtained via Bayesian network meta-analysis (Markov chain Monte Carlo simulation on the vague priors random-effects method for “bad” outcomes and zero values correction) with calculation of SUCRA value corresponding to all assessed doses, after verifying convergence (Brooks-Gelman-Rubin method) and inconsistency, using the NetMetaXL software (Canadian Agency for Drugs and Technologies in Health and Cornerstone Research Group) (14). Previously, all potassium-binding polymers as a whole were evaluated via pairwise meta-analysis (Mantel-Haenszel random-effects method) after verifying heterogeneity (χ^2^, *I*^2^) and the presence of reporting bias (visual inspection of funnel plots and calculation of Egger's test value), using the Review Manager software (RevMan) version 5.3 (Cochrane Collaboration) and META-analysis package FOr R (METAFOR) version 2.4 (R project).

In the second-step Bayesian network meta-analysis using the same mathematical assumptions as in the first-step network meta-analysis, calculations present the effects from binding potassium considering the outcome of increase in the dosing of spironolactone up to 50 mg/day in normokalemic patients with HF or resistant hypertension (who were also receiving other RAASi).

Recommendations of the Centre for Reviews and Dissemination (University of York) were followed in qualitative synthesis, that is, to assess all skewed and non-quantitative data (15).

## Results

This meta-analysis presents data on 2,279 individuals having participated in 11 clinical trials, of which 82.2% (*n* = 1,873), 63.5% (*n* = 1,447), 41.5% (*n* = 946), and 19.4% (*n* = 412) were, respectively, under treatment with HKID (e.g., RAASi, β-blockers, heparin, etc.), presented history of DM, had HF, or had resistant hypertension (16–40). Of the study participants, 79.2% (*n* = 1,805) had CKD into KDIGO GFR categories G3a–G5 and, the rest, NKF/early CKD (KDIGO GFR categories G1 and G2).

All participants in the included trials were either affected by or were at risk of developing hyperkalemia. However, with the exception of AMBER that studied only resistant hypertension patients (40), the rest of CT included patients with HF and other conditions (i.e., patients without known HF but with DM, and patients without HF but taking RAASi and other HKID were included in these trials). Dialysis patients and KTR did not participate in the included trials. Characteristics of the study population and key study details, in addition to the interventions, comparators, and outcomes, are available for readers online (Supplementary Table 1).

The pharmaceutical industry sponsored all these 11 CT, of which eight consisted in the phase 1–3 evaluation of the efficacy of patiromer (16–29), zirconium cyclosilicate (30–35), and sodium polystyrene sulfonate (SPS) (36) to treat hyperkalemia. Hyperkalemic patients may have HF or resistant hypertension or may present other causes for their hyperkalemia (*n* = 1,801): CKD into KDIGO GFR categories G3a–G5, DM under treatment with insulin and other antidiabetic drugs, or treatment with RAASi and other HKID. The impact of binding potassium as a treatment agent was measured on dichotomous and continuous outcomes centered on the influence on serum potassium levels.

Five phase 2 and 3 trials out of the retrieved 11 CT evaluated patiromer to prevent hyperkalemia, that is, for maintaining normokalemia (sK+ 3.5 to 5.0 mEq/L). Prevention of hyperkalemia was evaluated in these five trials as the impact of facilitating optimal dosing of spironolactone in schemes based on RAASi used by normokalemic patients, who had HF or resistant hypertension, with/without CKD into KDIGO GFR categories G3a to G5 (*n* = 1,135) (21–29, 37–40). Nevertheless, prevention of hyperkalemia was also measured on continuous outcomes concerning the influence on serum potassium levels.

Two CT out of the five CT on prevention of hyperkalemia, OPAL-HK (21–27) and AMENTHYST-DN (28, 29), were intended to assess patiromer also to treat hyperkalemia. Thus, six CT evaluated binding potassium exclusively to treat hyperkalemia (16–20, 30–36).

Trials included in this analysis were different concerning study design. OPAL-HK (21–27) and AMENTHYST-DN (28, 29) stratified study participants with hyperkalemia into the groups of mild hyperkalemia (sK+ <5.5 mEq/L) and moderate-to-severe hyperkalemia (sK+ <6.5 mEq/L) (21–29), and AMENTHYST-DN (28, 29) stratified participants with normokalemia into the groups of losartan 100 mg/day plus spironolactone and other RAASi plus spironolactone (28, 29). OPAL-HK (21–27), AMENTHYST-DN (28, 29), ZS-003/ZS-005 (31–33), and HARMONIZE (34, 35) were two-stage analyses. OPAL-HK, AMENTHYST-DN, and HARMONIZE contained single-arm explorations considering pre-treatment state as comparison (21–29, 34, 35). AMENTHYST-DN, ZS-003/ZS-005 and HARMONIZE were continued by extension follow-up studies (29, 32, 33, 35).

This analysis assessed two single-arm trials (16, 17, 37, 38) together with randomized trials controlled with placebo (21–27, 31–36, 39, 40) or with active comparisons consisting of standardized dietary restriction (18–20) or low doses of the potassium-binding polymers used as intervention (28, 29, 31–33).

**Figure 1:**
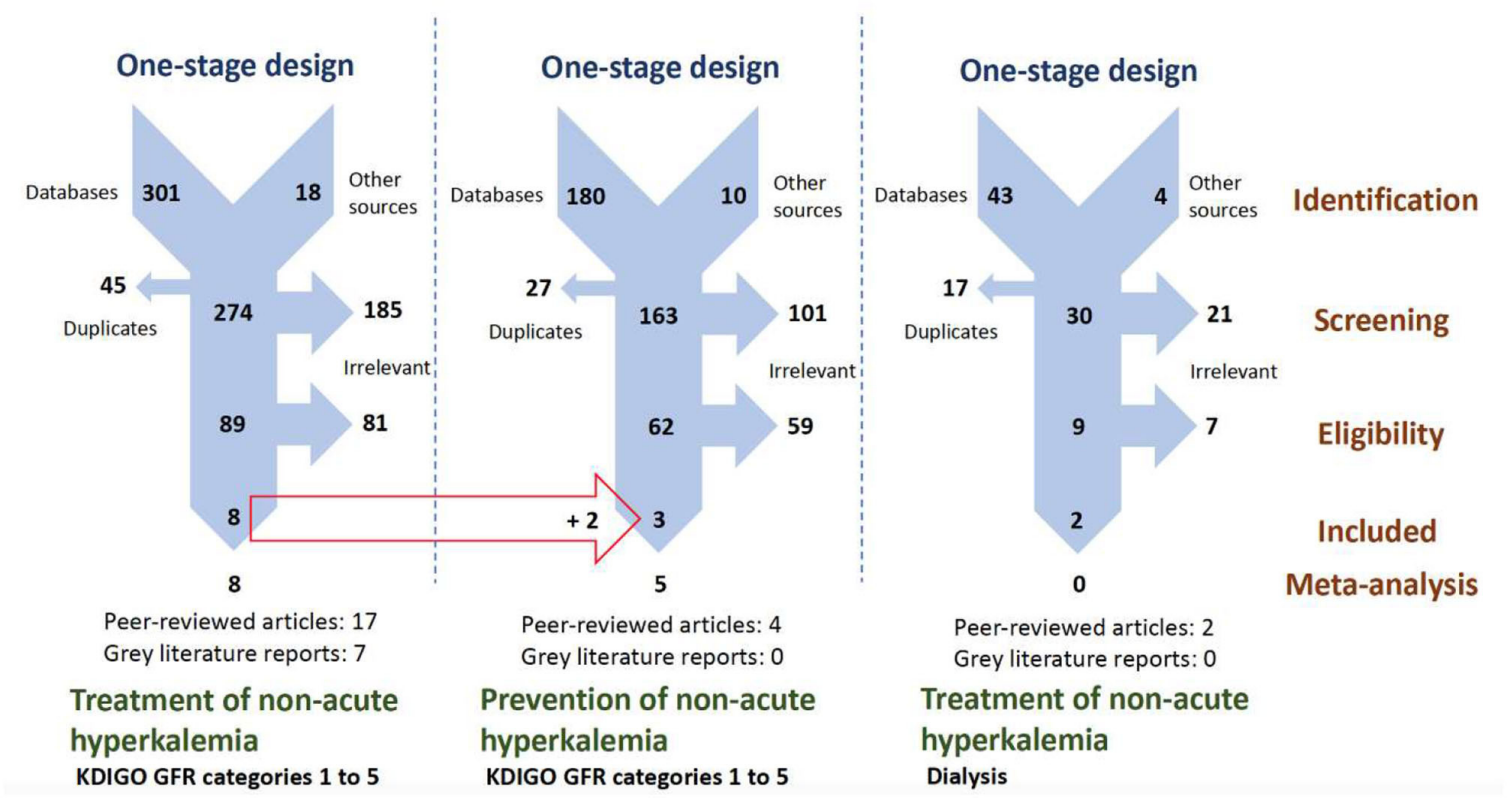
PRISMA flowcharts presenting our parallel one-stage systematic review selection process for retrieving CT providing mathematical data on treatment and prevention of hyperkalemia using potassium-binding polymers. CT, clinical trial; GFR, glomerular filtration rate; KDIGO, kidney disease–improving global outcomes; PRISMA, preferred reporting items for systematic reviews and meta-analyses.

Figure 1 presents the selection procedure followed that takes into account gray literature sources and allow the inclusion of seven unpublished reports (oral or posted communications) that provided important information from the published studies that were retrieved (17, 24–27, 32, 33). Gray literature did not provide new trials in addition to those retrieved from published sources.

Exclusions for this meta-analysis consisted of editorials and other opinion reports, observational studies that included real-world data evidence, and pre-clinical and clinical trials that did not evaluate our eligible outcomes. However, three and two trials studying calcium polystyrene sulfonate (CPS) and SPS in, respectively, CKD patients into KDIGO GFR categories G3a to G5 (41–43) and dialysis patients, were found (44, 45) but excluded as these CT did not provide the type of numerical data for our planned dichotomous analysis.

### First-Step Quantitative Analysis

Mathematical findings presented here came from moderate- to high-quality studies. Full assessment of risk of bias in the assessed CTs are consultable for readers online (Supplementary Table 2).

**Figure 2:**
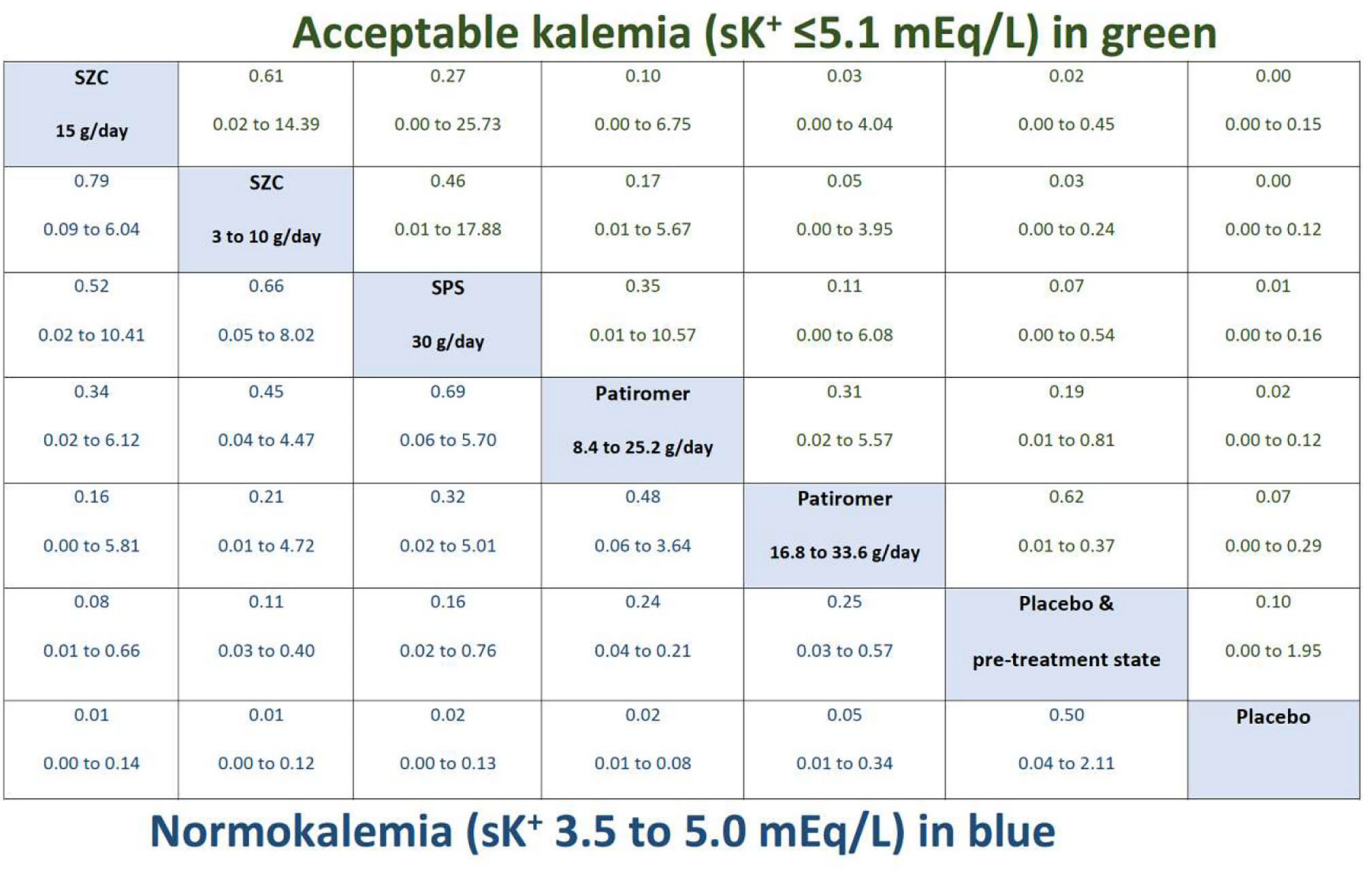
League table showing effect estimates in OR and 95% CrI for the outcomes of normokalemia (sK+ 3.5 to 5.0 mEq/L) and acceptable kalemia (sK+ ≤ 5.1 mEq/L) corresponding to all evaluated potassium-binding polymers for each possible (direct and indirect) comparison. CrI, credible intervals; OR, odds ratio; SPS, sodium polystyrene sulfonate; SZC, sodium zirconium cyclosilicate.

Figure 2 presents the league table from multiple-treatment meta-analysis calculations with eight CT studying hyperkalemic patients (*n* = 1,722) and shows significant positive effects compared to placebo according to the values of ORs and their corresponding 95% CrIs considering normokalemia (sK+ 3.5 to 5.0 mEq/L) and acceptable kalemia (sK+ ≤ 5.1 mEq/L) for patiromer 8.4–25.2 g/day (OR/95% CrI: 0.02/0.01–0.08, 0.02/0.00–0.12) (16–29), patiromer 16.8–33.6 g/day (0.05/0.01–0.34, 0.07/0.00–0.29) (16–29), SZC 15 g/day (0.01/0.00–0.14, 0.00/0.00–0.15) (30–35), SZC 3–10 g/day (0.01/0.00–0.12, 0.00/0.00–0.12) (30–35), and SPS 30 g/day (0.02/0.00–0.13, 0.01/0.00–0.16) (36). Size effects corresponding to such doses did not change when placebo and pre-treatment state were considered together.

The network level forest plot with estimates done on vague prior random-effects (Supplementary Figure 1) that presents a more comprehensive analysis extending our results at the pairwise level (data not shown) confirms effects from the assessed potassium-binding polymers and doses.

Low inconsistency was perceivable at the network level (Supplementary Figure 2), probably as a consequence of statistical heterogeneity observed both in pairwise and network calculations (*I*^2^ >65%). Risk for reporting bias [Egger's test (*t*)/degrees of freedom (df)/*p*: −2.9135, 21, <0.0001] was also important.

On the basis of the surface under the cumulative ranking area (SUCRA), there were differences between SZC (SUCRA >0.78), patiromer (SUCRA >0.58), and SPS (SUCRA <0.39) regarding their effects on the assessed outcomes. Indeed, mathematically, these molecules were different with respect to their capacity to achieve normokalemia and acceptable kalemia. However, such differences were more perceivable between either SZC or patiromer compared to SPS, so SZC and patiromer, respectively, were the best interventions against hyperkalemia (Table 1).

**Table 1:** SUCRA-based ranking of the evaluated commercial potassium-binding polymers.

**Drug intervention^†^**	**SUCRA^‡^**
**Potassium-binding polymers**	**Outcomes: A^**§**^/B^**&**^**
SZC 15 g/day	0.8294/0.8524
SZC 3–10 g/day	0.7830/0.8025
Patiromer 8.4–25.2 g/day	0.6832/0.6959
Patiromer 16.8–33.6 g/day	0.5797/0.5348
SPS 30 g/day	0.3890/0.3557
Placebo	0.1317/0.1510
Pre-treatment state	0.0041/0.0177

§ *Normokalemia (sK^+^ 3.5–5.0 mEq/L) and*

& *acceptable kalemia (sK^+^ ≤ 5.1 mEq/L) were the outcomes assessed at network level*.

† *The commercial potassium-binding polymers analyzed were ranked according to probabilities for being the best, the second best, the third best, and so on P(v = b), b = 1, …, a following Markov chain Monte Carlo methods*.

‡ *SUCRA for each polymer v out of the a competing polymers requires calculation of the a vector of the cumulative probabilities cum_v, b_ to be among the b best drug, b = 1, …, a. SPS, sodium polystyrene sulfonate; SUCRA, surface under the cumulative ranking area; SZC, sodium zirconium cyclosilicate*.

**Figure 3:**
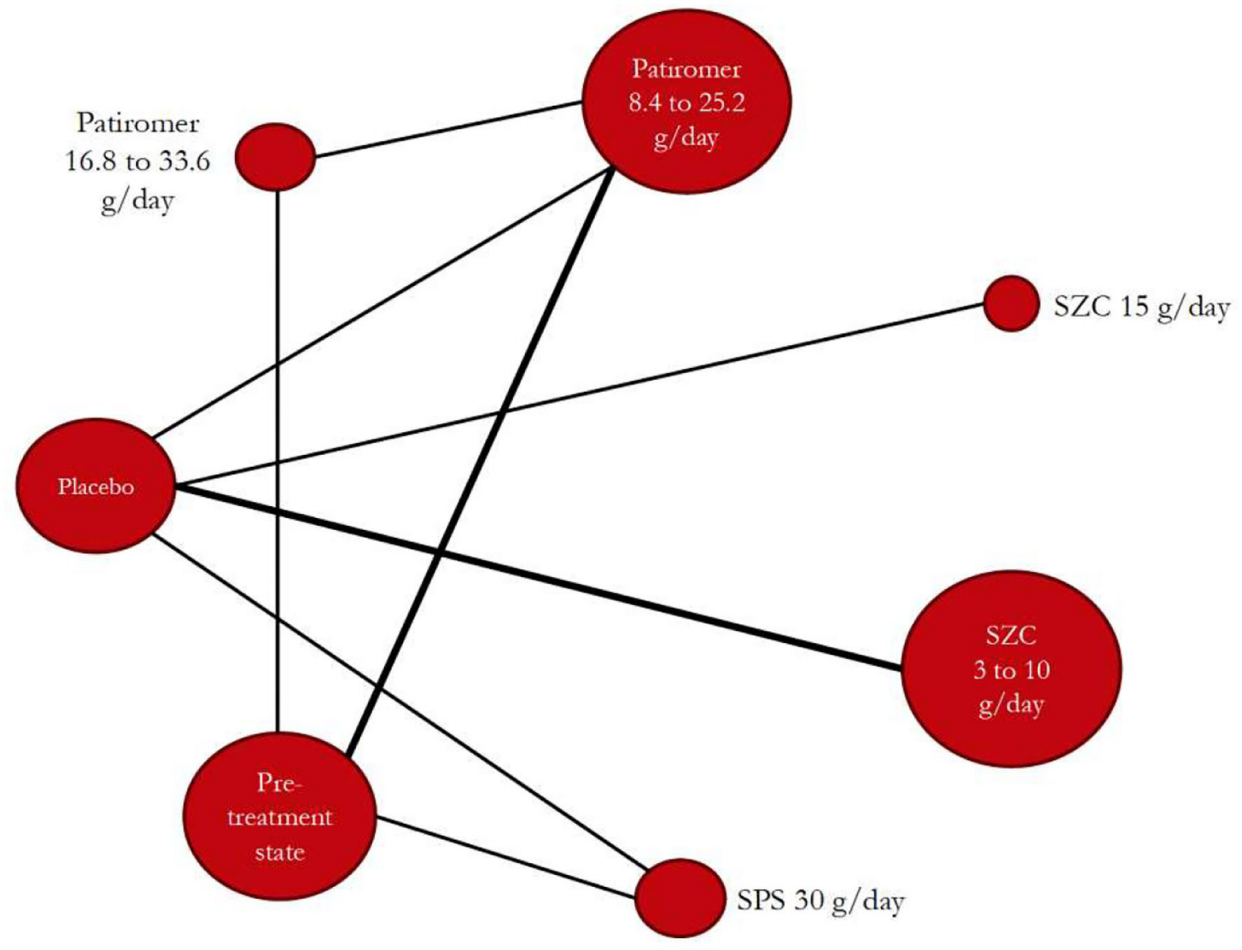
Bayesian network diagram constructed with the retrieved trials after searches and considering all evaluated potassium-binding polymers (nodes) and with showing all comparisons (lines between nodes). SPS, sodium polystyrene sulfonate; SZC, sodium zirconium cyclosilicate.

Figure 3 shows the Bayesian network diagram corresponding to the main analysis that shows the limited size of CT evidence, both in comparison density (thickness of lines according to the number of CTs in each comparison) and into the evidence for each potassium-binding polymer evaluated (node size according to the number of participants undergoing each polymer).

### Second-Step Quantitative Analysis

**Figure 4:**
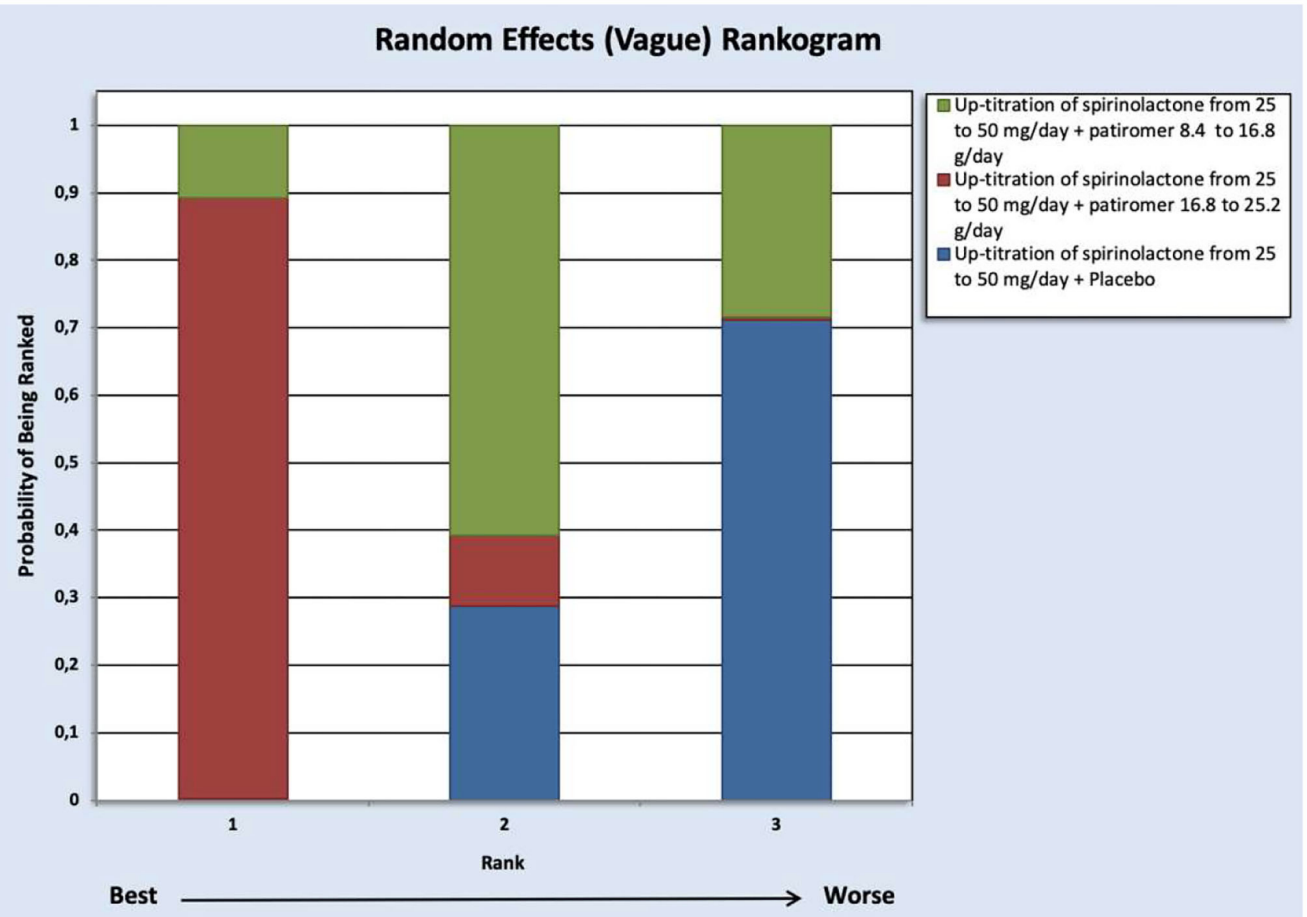
Vague priors random-effects rankogram for the evaluated patiromer doses allowing spironolactone up-titration up to 50 mg/day in subjects with HF or resistant hypertension (who may have or not CKD into KDIGO GFR categories G3a to G5, and were also receiving other RAASi). CKD, chronic kidney disease; GFR, glomerular filtration rate; KDIGO, kidney disease–improving global outcomes; HF, heart failure; RAASi, renin-angiotensin-aldosterone system inhibitors.

As depicted in Figure 4, according to the SUCRA value, the dose of spironolactone can be increased up to 50 mg/day in normokalemic patients with HF or resistant hypertension (who may have or not CKD into KDIGO GFR categories G3a–G5, and were also receiving other RAASi), when such individuals (*n* = 1,044) were treated with patiromer 16.8–25.2 g/day (SUCRA=0.94) or patiromer 8.4–16.8 g/day (SUCRA = 0.41). However, the dose of spironolactone cannot be increased when patiromer was not used (SUCRA = 0.15) (21–29, 37–40).

There were no data on SZC to perform calculations as those presented in Figure 4 for patiromer.

## Discussion

### Important Messages

According to our findings, there were differences between potassium-binding polymers concerning their capacity to achieve normal or acceptable serum potassium levels in individuals with hyperkalemia (with independence to the safety profile of the studied molecules). When normokalemia was achieved, as a measurable benefit for those using RAASi, current evidence shows association between the use of patiromer for maintaining normokalemia (i.e., as prevention drug against hyperkalemia) and optimal dosing of spironolactone (an increase of up to 50 mg/day) in subjects with HF or resistant hypertension using RAASi and needing the addition of an MRA. Nevertheless, for the moment, such an association for the use of zirconium cyclosilicate was not assessable. The lack of evidence should thus promote more research destined to confirm benefits from this new polymer in order to enlarge the armamentarium for managing patients affected by HF or resistant hypertension. These patients are commonly affected by CKD. In any case, all those needing treatment with RAASi who may be potentially at risk for hyperkalemia (e.g., patients with DM who may need treatment for proteinuria, patients with early hypertension, etc.) may also be included when assessing benefits for binding potassium.

This manuscript gathers and presents clinical trial evidence on the effectiveness of binding potassium. To our knowledge, this is the first dichotomous effects meta-analysis carried out on potassium-binding polymers that confirms findings from other summaries presenting data on conventional continuous outcomes (46–49). With a pharmacometrical perspective, the independent effects from the studied polymers and allowed doses are presented, in addition to the impact on optimizing treatment with RAASi requested by clinicians (50). In any case, even if more research is necessary to discern between benefits among all type of hyperkalemic patients, this study provides hard arguments to improve physicians' decision-making against this situation.

Hyperkalemia is associated with poorer clinical outcomes (51). Mainly caused by using RAASi and other hyperkalemia-inducing drugs such as β-blockers, heparin, etc. (52), hyperkalemia is more common in individuals with HF or resistant hypertension and CKD, especially when having DM (53–55). Therefore, by maintaining normokalemia, potassium-binding polymers may contribute to achieve better outcomes among those necessitating treatment with RAASi. In this sense, our findings show that hyperkalemia related to treatment by the combination of ACEi and/or ARBs with spironolactone is countered efficaciously by patiromer, and probably by zirconium cyclosilicate. Importantly, our analysis did not consider eplerenone, although its potential to induce hyperkalemia is probably similar to spironolactone (56–59), nor the first-in-class angiotensin receptor–neprilysin inhibitor (ARNI) sacubitril/valsartan that may be associated to moderate hyperkalemia in the cases of schemes including an MRA (60).

Hyperkalemia is a complication proper of CKD, as the kidneys are the main regulators of potassium homeostasis (61). In subjects with HF, DM, and in all those needing treatment with RAASi, potassium excretion is impaired in a non-acute manner, such requiring dietary restriction and pharmacological interventions (e.g., potassium-binding polymers, use of drugs with improved hyperkalemic profile, avoidance of other drugs increasing serum potassium levels, etc.) (62). Therefore, given the known safety profile of new polymers, their efficacy against hyperkalemia should from now on be taken into account and, particularly, the benefit elucidated here for patiromer. Such effectiveness is pending to be clarified for SZC.

The benefit of using patiromer will likely to be extended to SZC. Sodium zirconium cyclosilicate was authorized for use by the US Food and Drug Administration (FDA) and the European Medicine Agency (EMA) in 2018. Nevertheless, trial data on this new polymer as those already existing on patiromer are necessary. This analysis is, thus, a starting point and an inspiration of further evaluations on the benefit of these and other nanomolecules with interest by their profile to improve clinical outcomes. In any case, for the moment, results from the ongoing trial DIAMOND (NCT03888066) studying patiromer in 2,388 participants are expected, as they will contribute to clarify findings presented here. Regular updating of analyses like this is very important.

### Strengths and Limitations

Current recommendations to perform systematic reviews (63) and the standards to present optimally findings from network meta-analysis (64) proposed by the PRISMA research group were followed (14, 15). A multiple parallel one-stage systematic review design was used to retrieve dichotomous data on the studied outcomes. Prospective updating of the three systematic review protocols registered at the International Prospective Register of Systematic Reviews PROSPERO guarantees the transparency of all our methods and the entire analysis (65), confirming non-duplicity of the evaluation (66), and preventing the apparition of undesirable reporting biases (67).

Therefore, conclusions from this first-published dichotomous data pooling analysis can provide strong arguments on the kindness of the drugs assessed. Nevertheless, prudence is requested to readers when interpreting all messages transmitted here, as various limitations should be honestly declared. For the interest of clinicians, there were no data on SZC to perform calculations as those presented for patiromer in order to clarify a clinical impact of SZC on better dosing of MRA. More research will thus provide clinicians of more tools to combat hyperkalemia. Even if strength is the systematic approach, proper limitations of all systematic reviews should also be mentioned. Heterogeneity is an important limitation (66). Hyperkalemia was the common denominator of this analysis, leading to present effect sizes considering patients not only affected by HF or resistant hypertension; however, heterogeneity leads to present a benefit of binding potassium only for a subgroup of 1044 individuals with either HF or resistant hypertension (not for all patients with or at risk for hyperkalemia). A low inconsistency may influence in some degree the reliability of findings presented here, even if it is probably a reflection of heterogeneity (68). Publication bias was also observed and measured, and it is a discouraging finding, leading to overly optimistic conclusions in a meta-analysis (69): It is important to note, however, that our assessments include trials of <1,000 participants, so this study contributes to clarify potential false substantial effects reported by small trials (70). Furthermore, restricted evidence was an important problem to perform calculations that may be noted in effect sizes of overlapping doses of patiromer. In this context, the results obtained in our analysis, considering the outcome definitions that are in accordance to established limits in guidelines and studies (71–80), may change when performing calculations that consider other upper limits of normal (ULN) for serum potassium levels. Finally, non-assessable data on CPS involving patients before dialysis and on CPS and SPS involving patients undergoing dialysis may be considered also as arguments of restricted evidence.

### Research Opportunities

The potential of zirconium cyclosilicate should be explored further, as no data exist to assess properly its capacity to optimize the dosing of RAASi, contrarily as it occurs for patiromer. In any case, considering their safety profile, new potassium-binding polymers may be considered as clinically relevant nanomolecules. In this way, regular intervals updating of evidence will be particularly important to improve the treatment of susceptible patient populations as those involved in this study, which may include observational evidence, if required (81).

Findings presented here correspond only to analyses carried out on clinical trials. This study belongs to an ongoing project aiming to assess clinical trial evidence and observational real-life evidence on the effectiveness of all commercial potassium-binding polymers, as performed by our team in a previous project (82), so further results are pending to be presented.

## Conclusions

This manuscript presents findings from the first dichotomous data pooling analysis on the effectiveness of binding potassium. The assessed molecules were different with respect to their capacity to achieve normokalemia (sK+ 3.5–5.0 mEq/L) and acceptable kalemia (sK+ ≤ 5.1 mEq/L) in individuals with hyperkalemia (sK+ >5.1 mEq/L), and current evidence shows that patiromer can lead to the optimal dosing of spironolactone (and probably of other MRA) into schemes based on RAASi used by individuals with HF or resistant hypertension. These patients commonly have CKD. For the moment, there is no evidence to conclude that using zirconium cyclosilicate may allow optimizing treatment with MRA in RAASi-based schemes needing these patients. The lack of evidence should thus promote more research destined to confirm the benefits from this new polymer in order to enlarge options to control hyperkalemia. More research is also necessary to discern between the benefits of binding potassium among all type of hyperkalemic patients, for example, patients with DM who may need treatment for proteinuria, patients with early hypertension, etc. Future meta-analyses for updating findings presented here require more research for more homogenous findings on interventions against hyperkalemia.

## Data Availability Statement

The original contributions presented in the study are included in the article/Supplementary Material, further inquiries can be directed to the corresponding author/s.

## Author Contributions

EG-A, FH-G, FÁ, and FL-S developed the hypothesis and study design. ÁM-B-M, ÁN-R, DF-Z, DM-G, EB-M, EG-A, FP, FL-S, JB-M, JG-V, MC, and MP performed the literature searches and/or screened papers. CO-S, FH-G, FÁ, and MC performed the data analyses. All authors contributed to the drafting and critical revision of all manuscript versions.

## Conflict of Interest

The authors declare that the research was conducted in the absence of any commercial or financial relationships that could be construed as a potential conflict of interest.

## Publisher's Note

All claims expressed in this article are solely those of the authors and do not necessarily represent those of their affiliated organizations, or those of the publisher, the editors and the reviewers. Any product that may be evaluated in this article, or claim that may be made by its manufacturer, is not guaranteed or endorsed by the publisher.
