# Supplementary material for: Binding Potassium to Improve Treatment With Renin-Angiotensin-Aldosterone System Inhibitors: Results From Multiple One-Stage Pairwise and Network Meta-Analyses of Clinical Trials
Source: Front Med (Lausanne). 2021 Aug 19;8:686729. doi: 10.3389/fmed.2021.686729 (PMC8416895; doi:10.3389/fmed.2021.686729)
Supplement: Supplementary file 1 [file Data_Sheet_1.pdf]

Supplementary material of the article:

Binding Potassium to Improve Treatment With Renin-Angiotensin-Aldosterone System Inhibitors: Results From Multiple One-Stage Pairwise and Network Meta-analyses of Clinical Trials

Frank Lizaraso-Soto, Eduardo-Gutiérrez-Abejón, Juan Bustamante-Munguira, Débora Martín-García, María Montserrat Chimeno, Álvaro Nava-Rebollo, Álvaro Maurtua-Briseño-Meiggs, Darío Fernández, Elena Bustamante-Munguira, Félix Jesús de Paz, Jesús Grande-Villoria, Carlos Ochoa-Sangrador, Manuel Pascual, F. Javier Álvarez, Francisco Herrera-Gómez

Table S1. Participants, interventions, comparators, and outcomes in the mathematically assessed trials, that aimed evaluation of (a) treatment of hyperkalemia, (b) prevention of hyperkalemia.

Table S2. Risk of bias assessment in the included studies.

Figure S1. Network forest plot of all possible comparisons with potassium-binding polymers, placebo and pre-treatment state.

Figure S2. Random effects inconsistency plot of comparisons with potassium-binding polymers, placebo and pre-treatment state.

Table S1. Participants, interventions, comparators, and outcomes in the mathematically assessed trials, that aimed evaluation of:
(a) Treatment of hyperkalemia,

**Trial**<br>Details	**Design**	**Follow-up**<br>(in days)	**Participants/population**<br>Characteristics	**Interventions**<br>(n)	**Comparators**<br>(n)	**Outcomes**	**Co-interventions**
**Bushinsky et al.**<br>Eastern Europe<br>(1,2)	SAT	6	Mean age in yrs (SD), males (%), DM (%), HF (%), HKID (%): 58.7 (12.3), 60.0, 60.0, 28.0, 100.0.<br>KDIGO GFR G3a to G5/KRT (%): 88.0/0.0<br>Causes of CKD (%): NA.	Patiromer PO 8.4 g BID for 2 days (25).	Pre-treatment state (25).	Change in sK^+ from baseline / at time-points.<br>Patients achieving normokalemia (sK^+ 3.5 to 5.0 mEq/L) / acceptable kalemia (sK^+ until 5.1 to 5.4 mEq/L).<br>Time-to-effect.<br>TEAE.	Dietary restrictions.<br>RAASi, diuretics, β-blockers, insulin, and other antidiabetic drugs.
**TOURMALINE**<br>NCT02694744<br>USA (3–5)	RCT	28	Mean age in yrs (SD), males (%), DM (%), HF (%), HKID (%): 66.6 (11.9), 65.1, 82.1, 9.0, 58.9.<br>KDIGO GFR G3a to G5/KRT (%): 69.6/0.0<br>Causes of CKD (%): NA.	Patiromer PO 8.4 g QD with standardized dietary restriction for 28 days (57).	Patiromer PO 8.4 g QD without standardized dietary restriction for 28 days (57).	Patients achieving normokalemia (sK^+ 3.5 to 5.0 mEq/L) / acceptable kalemia (sK^+ until 5.1 to 5.4 mEq/L).<br>Between-group difference in mean sK^+.<br>Cations in blood.<br>TEAE.	RAASi, diuretics, β-blockers, insulin, and other antidiabetic drugs.
**OPAL-HK**<br>NCT01810939<br>USA, UE, and Eastern Europe<br>(6–12)	Two-stage ST-SAT plus ST-RCT	28 + 56	Mean age in yrs (SD), males (%), DM (%), HF (%), HKID (%): 64.2 (10.5), 58.0, 57.0, 42.0, 100.0.<br>KDIGO GFR G3a to G5/KRT (%): 90.9/0.0<br>Causes of CKD (%): NA.	Patiromer PO 4.2 or 8.4 g BID for 28 days (243), then 4.2 or 8.4 g BID plus RAASi for 56 days (55).	Patiromer PO 4.2 or 8.4 g BID for 28 days (243), then placebo plus RAASi for 56 days (52).	Change in mean sK^+ from baseline.<br>Patients achieving normokalemia (sK^+ 3.5 to 5.0 mEq/L) / acceptable kalemia (sK^+ until 5.1 to 5.4 mEq/L).<br>Time-to-hyperkalemia, time-	No dietary restrictions.<br>RAASi (including aldosterone antagonists), diuretics, and β-blockers.

						to-dose change in RAAS inhibitor.<br>TEAE.	
**AMETHYST-DN**<br>NCT01371747<br>Eastern Europe (13,14)	Two-stage ST-SAT plus ST-RCT and EXTFW	392 + 28	Mean age in yrs (SD), males (%), DM (%), HF (%), HKID (%): 66.3 (8.6), 63.2, 100.0, 34.5, 100.0.<br>KDIGO GFR G3a to G5/KRT (%): 88.2/0.0<br>Causes of CKD (%): NA.	Losartan PO 100 mg per day or another RAASi with spironolactone PO 25 or 50 mg QD for 28 days, then patiromer PO 8.4 g, 12.6 g, or 16.8 g BID for 365 days (222)	Losartan PO 100 mg per day or another RAASi with spironolactone PO 25 or 50 mg QD for 28 days, then patiromer PO 4.2 g, 8.4 g, or 12.6 g BID for 365 days (84)	Change in sK^+ from baseline / at time-points.<br>Patients achieving normokalemia (sK^+ 3.5 to 5.0 mEq/L) / acceptable kalemia (sK^+ until 5.1 to 5.4 mEq/L).<br>TEAE.	No dietary restrictions.<br>RAASi (including spironolactone).
**ZS-002**<br>NCT01493024<br>USA (15)	RCT	7	Mean age in yrs (SD), males (%), DM (%), HF (%), HKID (%): 71.1 (9.0), 55.8, 55.4, 10.8, 64.8.<br>KDIGO GFR G3a to G5/KRT (%): 69.4/0.0<br>Causes of CKD (%): NA.	SZC PO 0.3 g, 3 g, or 10 g TID for 2 to 4 days (60).	Placebo for 2 to 4 days (30).	sK^+ decline rate.<br>Between-group difference in sK^+ ERC.<br>Change in sK^+ from baseline / at time-points.<br>Patients achieving normokalemia (sK^+ 3.5 to 5.0 mEq/L) / acceptable kalemia (sK^+ until 5.1 to 5.4 mEq/L).<br>Cations and anions in blood and urine.<br>TEAE.	No dietary restrictions.<br>RAASi (including spironolactone), insulin, and other antidiabetic drugs.
**ZS-003**<br>NCT01737697<br>NCT02163499<br>USA, Australia, and South Africa (16–18)	Two-stage RCT and EXTFW	21 + 365	Mean age in yrs (SD), males (%), DM (%), HF (%), HKID (%): 65.7 (12.2), 59.5, 59.9, 39.8, 66.7.<br>KDIGO GFR G3a to G5/KRT (%): 74.5/0.0<br>Causes of CKD (%): NA.	SZC PO 1.25 g, 2.5 g, 5 g, or 10 g TID for 2 days, then 1.25 g, 2.5 g, 5 g, or 10 g QD for 12 days (595), and thereafter 10 g TID for 1 to 3 days plus 5 g QD	Placebo for 2 days, then SZC PO 1.25 g or 2.5 g QD for 12 days (158), and thereafter 10 g TID for 1 to 3 days plus 5 g QD for 365 days	Between-group difference in sK^+ ERC / mean sK^+.<br>Patients achieving normokalemia (sK^+ 3.5 to 5.0 mEq/L) / acceptable kalemia (sK^+ until 5.1 to 5.4 mEq/L).	No dietary restrictions.<br>RAASi, diuretics, insulin, and other antidiabetic drugs.

				for 365 days (possibility for up-titration up to 15 mg per day).	(possibility for up-titration up to 15 mg per day).	Time-to-normokalemia, time-to-increase sK^+ 0.5 mmol/L, time-to-decrease sK^+ 0.5 mmol/L.<br>TEAE.<br>Various surrogate markers.	
**HARMONIZE**<br>NCT02088073<br>NCT02107092<br>USA, Australia, and South Africa (19,20)	Two-stage SAT plus RCT and EXTFW	2 + 28 + 335	Mean age in yrs (SD), males (%), DM (%), HF (%), HKID (%): 63.7 (12.9), 58.8, 66.3, 37.4, 68.9.<br>KDIGO GFR G3a to G5/KRT (%): 69.4/0.0<br>Causes of CKD (%): NA.	SZC PO 10 g TID for 2 days, then 5 g, 10 g, or 15 g QD for 28 days (152), and thereafter (only some participants) 10 g QD for 335 days (123).	Placebo for 28 days (85), then (only some participants) SZC PO 10 g QD for 335 days (123).	Between-group difference in mean sK^+.<br>Patients achieving normokalemia (sK^+ 3.5 to 5.0 mEq/L) / acceptable kalemia (sK^+ until 5.1 to 5.4 mEq/L).<br>Time-to-hyperkalemia.<br>Normokalemia cumulative-days.<br>Mean intra-subject sK^+ SD.<br>Change in aldosterone and renin levels in blood.<br>TEAE.<br>Various surrogate markers.	No dietary restrictions.<br>Discontinuation of RAASi.
**SKIP**<br>NCT02065076<br>Canada (21)	RCT	7	Mean age in yrs (SD), males (%), DM (%), HF (%), HKID (%): 72.3 (10.6), 69.5, 72.7, 18.8, 94.0.<br>KDIGO GFR G3a to G5/KRT (%): 100.0/0.0<br>Causes of CKD (%): vascular (87.5), ADPKD (6.3), other (6.2).	SPS PO 30 g QD for 7 days (16).	Placebo for 7 days (17).	Between-group difference in mean sK^+.<br>Patients achieving normokalemia (sK^+ 3.5 to 5.0 mEq/L) / acceptable kalemia (sK^+ until 5.1 to 5.4 mEq/L).	Dietary restrictions.<br>RAASi and insulin.

						Cations and anions in blood.<br>TEAE.	

(b) Prevention of hyperkalemia.

**Trial**<br>Details	**Design**	**Follow-up**<br>(in days)	**Participants/population**<br>Characteristics	**Interventions**<br>(n)	**Comparators**<br>(n)	**Outcomes**	**Co-interventions**
**Patiromer-204**<br>NCT01130597<br>Eastern Europe<br>(22,23)	SAT	56	Mean age in yrs (SD), males (%), DM (%), HF (%), HKID (%): 70.8 (8.5), 61.9, 42.9, 100.0, 100.0.<br>KDIGO GFR G3a to G5/KRT (%): 100.0/0.0<br>Causes of CKD (%): vascular (57.2), unknown (30.2), DM (6.3), urologic/congenital (6.3).	Spironolactone PO 25 or 50 mg QD plus Patiromer PO up to 50.4 g QD for 56 days (63).	Pre-treatment state with spironolactone not started (63).	Successful spironolactone up-titration up to 50 mg per day.<br>Patients maintaining normokalemia (sK^+ 3.5 to 5.0 mEq/L) / acceptable kalemia (sK^+ until 5.1 to 5.4 mEq/L).<br>Patients requiring patiromer up/down-titration.<br>Patiromer mean-dose.<br>TEAE.	Dietary restrictions.<br>Prohibition of K^+- and PO_4^{3-}-binding polymers, and other K^+-sparing drugs.<br>RAASi, diuretics, and β-blockers.
**PEARL-HF**<br>NCT00868439<br>USA, UE, Eastern Europe, and Russia (24)	RCT	28	Mean age in yrs (SD), males (%), DM (%), HF (%), HKID (%): 68.0 (10.0), 60.6, 31.7, 100.0, 100.0.<br>KDIGO GFR G3a to G5/KRT (%): 26.9/0.0<br>Causes of CKD (%): NA.	Spironolactone PO 25 or 50 mg QD plus Patiromer PO 16.8 g BID for 28 days (60).	Spironolactone PO 25 or 50 mg QD plus placebo for 28 days (60).	Change in mean sK^+ from baseline.<br>Successful spironolactone up-titration up to 50 mg per day.<br>Patients not maintaining normokalemia (sK^+ 3.5 to 5.0 mEq/L) / acceptable kalemia (sK^+ until 5.1 to 5.4 mEq/L).<br>TEAE.	Dietary restrictions.<br>Prohibition of K^+- and PO_4^{3-}-binding polymers, and other K^+-sparing drugs.<br>RAASi, diuretics, and β-blockers.

**AMBER** NCT03071263 USA, UK, UE, Eastern Europe, and South Africa (25)	RCT	112	Mean age in yrs (SD), males (%), DM (%), HF (%), HKID (%): 68.2 (11.7), 52.0, 49.5, 45.0, 100.0.<br>KDIGO GFR G3a to G5/KRT (%): 100.0/0.0<br>Causes of CKD (%): NA.	Spironolactone PO 25 mg QD plus Patiromer PO 8.4 g QD for 84 days (148).	Spironolactone PO 25 mg QD plus placebo for 84 days (147).	Between-group difference in patients remaining on spironolactone / SBP / spironolactone cumulative-dose.<br>Patients not maintaining normokalemia (sK^+ 3.5 to 5.0 mEq/L) / acceptable kalemia (sK^+ until 5.1 to 5.4 mEq/L).<br>Change in 7-day SBP / albuminuria.<br>Quality of life.<br>TEAE.	RAAS inhibitors, diuretics, and β-blockers.

Abbreviations: ADPKD, autosomal-dominant polycystic kidney disease; AMBER, A Randomized, Double-Blind, Placebo Controlled, Parallel Group Study of Patiromer for the Enablement of Spironolactone Use for Blood Pressure Control in Patients With Resistant Hypertension and Chronic Kidney Disease; AMETHYST-DN, A Multicenter, Randomized, Open-Label, Dose Ranging Study to Evaluate the Efficacy and Safety of Patiromer in the Treatment of Hyperkalemia in Patients With Hypertension and Diabetic Nephropathy Receiving Angiotensin-converting Enzyme Inhibitor (ACEI) and/or Angiotensin II Receptor Blocker (ARB) Drugs, With or Without Spironolactone; BID, twice a day; CKD, chronic kidney disease; CNI, calcineurin inhibitor; DM, diabetes mellitus; EXTFW, extension follow-up study; GFR, glomerular filtration rate; HARMONIZE, Multicenter, Multi-phase, Multi-dose, Prospective, Double-blind, Placebo-controlled, Maintenance Study of Safety and Efficacy of ZS (Microporous, Fractionated, Protonated Zirconium Silicate) in Hyperkalemia; HF, heart failure; HKID, hyperkalemia-inducing drugs; KDIGO; Kidney Disease: Improving Global Outcomes; KRT, kidney replacement therapy; mEq/L, milliequivalents per liter NA, non-available; OPAL-HK, A Two-Part, Single-Blind, Phase 3 Study Evaluating the Efficacy and Safety of Patiromer for the Treatment of Hyperkalemia; PEARL-HF, A Multicenter, Randomized, Double-blind, Placebo-Controlled, Parallel-Group, Multiple-Dose Study to Evaluate the Effects of Patiromer in Heart Failure Patients; PO, per os; QD, every day; RAASi, renin-angiotensin-aldosterone system inhibitors; RCT, randomized controlled trial; RLY5016-204, A Multicenter, Open-Label, Single-Arm Study to Evaluate a Titration Regimen for Patiromer in Heart Failure Patients With Chronic Kidney Disease; SAT, single-arm trials; SBP, systolic blood pressure; SD, standard deviation; sK^+, serum potassium level; SKIP, Efficacy of Sodium Polystyrene Sulfonate in the Treatment of Hyperkaliemia in Ambulatory Pre-dialysis Outpatients: A Randomized Triple-blind Placebo-controlled Trial; SPS, sodium polystyrene sulfonate; ST, stratified trial design; SZC, sodium zirconium cyclosilicate; TEAE, treatment-emergent adverse event; TID, three times a day; TOURMALINE, The Effect of Food: An Open-Label, Randomized, Parallel Group Phase 4 Study of the Efficacy and Safety of Patiromer for Oral Suspension With or Without Food for the Treatment of Hyperkalemia; ZS-002, Multicenter, Prospective, Randomized, Placebo-Controlled, Double-blind Dose Escalating Study of Safety, Tolerability and Pharmacodynamics of Zirconium Silicate in Chronic Kidney Disease and Moderate Kidney Dysfunction With Mild Hyperkalemia; ZS-003, Multicenter, Two-phase, Multi-dose, Prospective, Randomized, Double-blind, Placebo-Controlled Study of Safety and Efficacy of Microporous, Fractionated, Protonated Zirconium Silicate in Mild to Moderate Hyperkalemia.

Table 2. Risk of bias assessment in the included studies.

Trials	Random sequence generation	Allocation concealment	Blinding of participants and personnel	Blinding of outcome assessment	Incomplete outcome data	Selective reporting	Other bias
**Bushinsky et al.** (1,2)	H	H	U	U	U	U	U
**TOURMALINE** (3–5)	L	L	U	U	U	U	U
**OPAL-HK** (6–12)	U	L	L	L	L	L	L
**AMETHYST-DN** (13,14)	U	L	L	L	L	U	U
**ZS-002** (15)	L	L	L	U	L	L	U
**ZS-003** (16–18)	L	L	L	U	L	U	U
**HARMONIZE** (19,20)	U	L	U	U	L	U	U
**SKIP** (21)	L	L	L	U	U	L	U
**Patiromer-204** (22,23)	H	H	U	U	U	U	U
**PEARL-HF** (24)	L	L	U	U	U	U	U
**AMBER** (25)	L	L	L	U	L	L	L

The judgement for each of risk of bias domain is presented as (L), (U) or (H) to indicate low, unclear, or high risk of bias, respectively.

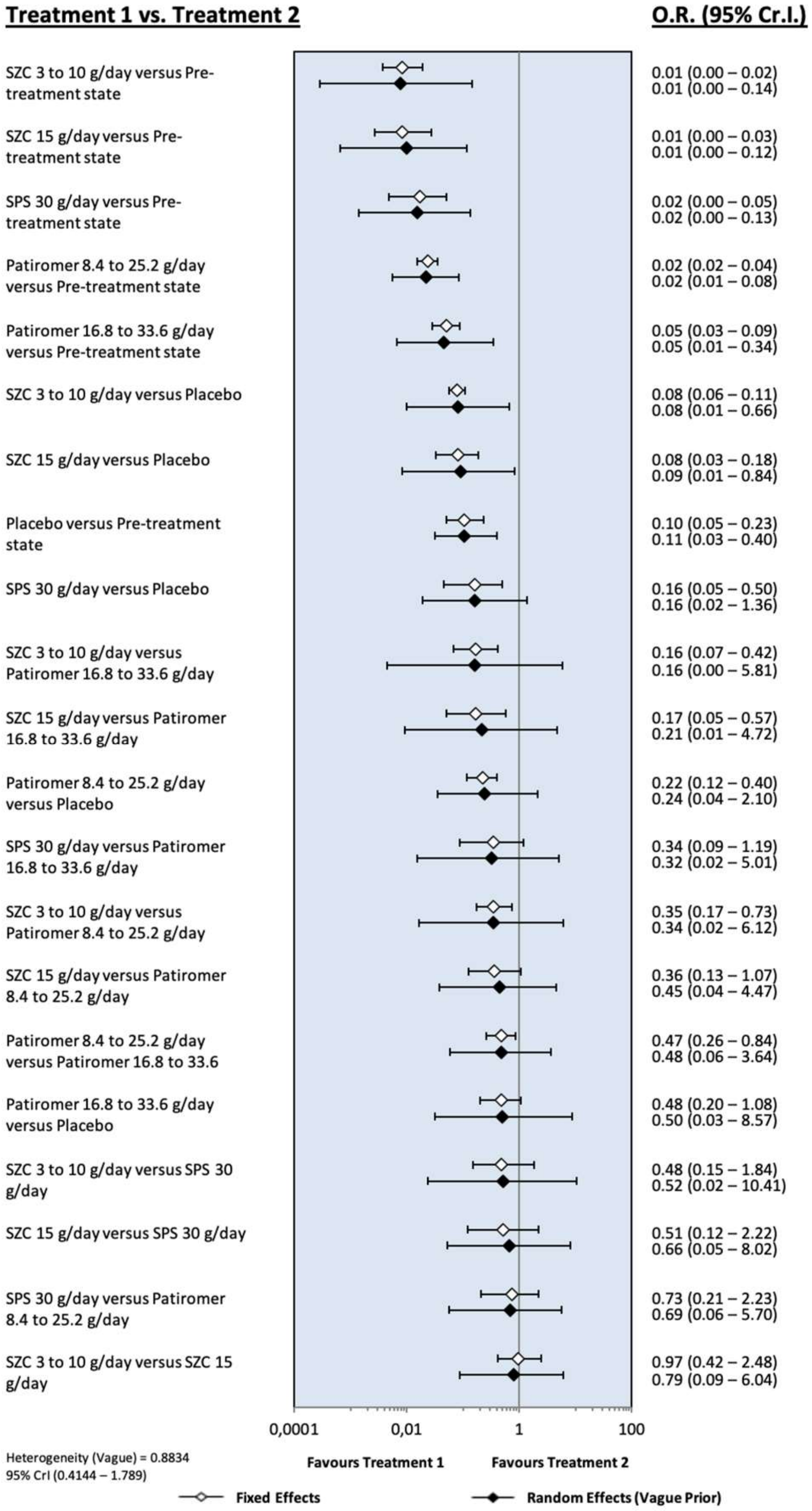

Figure S1. Network forest plot of all possible comparisons with potassium-binding polymers, placebo and pre-treatment state. CrI, credible intervals; OR, odds ratio.

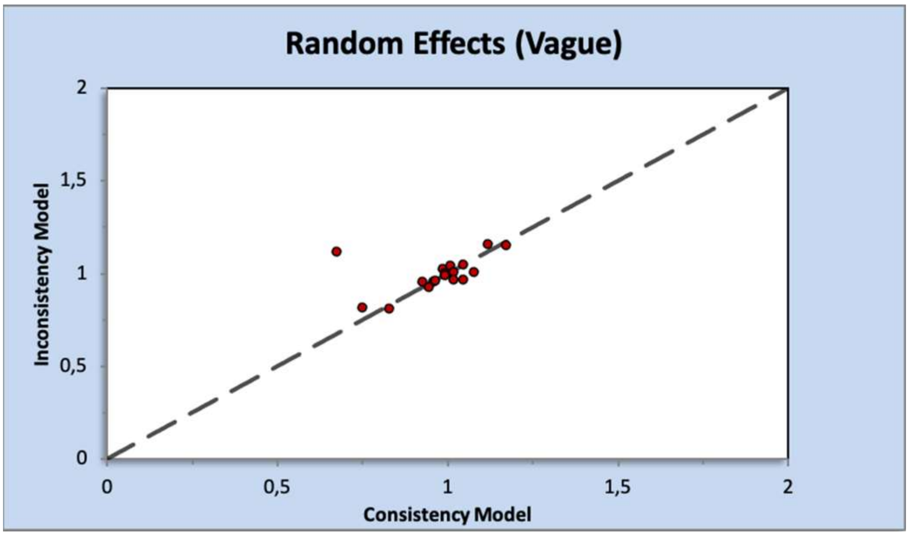

Figure S2. Random effects inconsistency plot of comparisons with potassium-binding polymers, placebo and pre-treatment state.

REFERENCES

1. Bushinsky, D.A.; Williams, G.H.; Pitt, B.; Weir, M.R.; Freeman, M.W.; Garza, D.; Stasiv, Y.; Li, E.; Berman, L.; Bakris, G.L. Patiromer induces rapid and sustained potassium lowering in patients with chronic kidney disease and hyperkalemia. *Kidney Int.* **2015**, *88*, 1427–1433. https://doi.org/10.1038/ki.2015.270
2. Bushinsky, D.A.; Bakris, G.L.; Williams, G.; Pitt, B.; Mayo, M.; Garza, D.; Stasiv, Y.; Li, E.; Berman, L. SA-PO153: Patiromer induced a rapid onset of action and sustained K+ lowering throughout the dosing period in CKD patients with hyperkalemia. *J. Am. Soc. Nephrol.* **2014**, *25*, 669A. https://www.asn-online.org/education/kidneyweek/archives/KW14Abstracts.pdf
3. Pergola, P.E.; Spiegel, D.M.; Warren, S.; Yuan, J.; Weir, M.R. Patiromer lowers serum potassium when taken without food: Comparison to dosing with food from an open-label, randomized, parallel group hyperkalemia study. *Am. J. Nephrol.* **2017**, *46*, 323–332. https://doi.org/10.1159/000481270
4. Bushinsky, D.A.; Spiegel, D.M.; Yuan, J.; Warren, S.; Fogli, J.; Pergola, P.E. Effects of the potassium-binding polymer patiromer on markers of mineral metabolism. *Clin. J. Am. Soc. Nephrol.* **2019**, *14*, 103–110. https://doi.org/10.2215/CJN.04500418
5. Kloner, R.A.; Gross, C.; Yuan, J.; Conrad, A.; Pergola, P.E. Effect of patiromer in hyperkalemic patients taking and not taking RAAS inhibitors. *J. Cardiovasc. Pharmacol. Ther.* **2018**, *23*, 524–531. https://doi.org/10.1177/1074248418788334
6. Weir, M.R.; Bakris, G.L.; Bushinsky, D.A.; Mayo, M.R.; Garza, D.; Stasiv, Y.; Wittes, J.; Christ-Schmidt, H.; Berman, L.; Pitt, B. Patients with kidney disease and hyperkalemia receiving RAAS inhibitors. *N. Engl. J. Med.* **2015**, *372*, 211–221. https://doi.org/10.2215/CJN.01541005
7. Weir, M.R.; Mayo, M.R.; Garza, D.; Arthur, S.A.; Berman, L.; Bushinsky, D.; Wilson, D.J.; Epstein, M. Effectiveness of patiromer in the treatment of hyperkalemia in chronic kidney disease patients with hypertension on diuretics. *J. Hypertens.* **2017**, *35*, S57–S63. https://doi.org/10.1097/HJH.0000000000001278
8. Pitt, B., Bakris, G.L.; Bushinsky, D.A.; Garza, D.; Mayo, M.R.; Stasiv, Y.; Christ-Schmidt, H.; Berman, L.; Weir, M.R. Effect of patiromer on reducing serum potassium and preventing recurrent hyperkalaemia in patients with heart failure and chronic kidney disease on RAAS inhibitors. *Eur. J. Heart Fail.* **2015**, *17*, 1057–1065. https://doi.org/10.1002/ejhf.402
9. Weir, M.R.; Bakris, G.L.; Bushinsky, D.A.; Mayo, M.; Garza, D.A.; Stasiv, Y.; Hou, Y.; Christ-Schmidt, H.; Berman, L. FR-PO792: Patiromer lowers serum K+ and prevents recurrent hyperkalemia in patients with diabetes and CKD on RAAS Inhibitors: Subgroup results. *J. Am. Soc. Nephrol.* **2014**, *25*, 550A–551A. https://www.asn-online.org/education/kidneyweek/archives/KW14Abstracts.pdf
10. Weir, M.R.; Bakris, G.L.; Bushinsky, D.A.; Mayo, M.; Garza, D.A.; Stasiv, Y.; Wittes, J.; Christ-Schmidt, H.; Berman, L. FR-PO810: Patiromer reduced RAASi dose discontinuations in CKD patients with moderate-to-severe hyperkalemia. *J. Am. Soc. Nephrol.* **2014**, *25*, 555A–556A. https://www.asn-online.org/education/kidneyweek/archives/KW14Abstracts.pdf
11. Weir, M.R.; Bushinsky, D.A.; Mayo, M.; Garza, D.A.; Stasiv, Y.; Wilson, D.J.; Arthur, S.; Bakris, G.L. TH-OR035: Patiromer lowers serum K+ and prevents recurrent hyperkalemia in CKD patients ≥65 years of age on RAAS inhibitors. *J. Am. Soc. Nephrol.* **2015**, *26*, 10A. https://www.asn-online.org/education/kidneyweek/archives/KW15Abstracts.pdf
12. Weir, M.R.; Mayo, M.; Garza, D.A.; Stasiv, Y.; Arthur, S.; Berman, L.; Bushinsky, D.A.; Wilson, D.J.; Epstein, M. TH-PO658: Chronic diuretic therapy does not impair the effectiveness of patiromer in hyperkalemic patients with CKD. *J. Am. Soc. Nephrol.* **2015**, *26*, 240A. https://www.asn-online.org/education/kidneyweek/archives/KW15Abstracts.pdf
13. Bakris, G.L.; Pitt, B.; Weir, M.R.; Freeman, M.W.; Mayo, M.R.; Garza, D.; Stasiv, Y.; Zawadzki, R.; Berman, L.; Bushinsky, D.A. Effect of patiromer on serum potassium level in patients with hyperkalemia and diabetic kidney disease: The AMETHYST-DN randomized clinical trial. *JAMA.* **2015**, *314*, 151–161. https://doi.org/10.1001/jama.2015.7446
14. Pitt, B.; Bakris, G.L.; Weir, M.R.; Freeman, M.W.; Lainscak, M.; Mayo, M.R.; Garza, D.; Zawadzki, R.; Berman, L.; Bushinsky, D.A. Long-term effects of patiromer for hyperkalaemia treatment in patients with mild heart failure and diabetic nephropathy on angiotensin-converting enzymes/angiotensin receptor blockers: results from AMETHYST-DN. *ESC Heart Fail.* **2018**, *5*, 592–602. https://doi.org/10.1002/ehf2.12292
15. Ash, S.R.; Singh, B.; Lavin, P.T.; Stavros, F.; Rasmussen, H.S. A phase 2 study on the treatment of hyperkalemia in patients with chronic kidney disease suggests that the selective potassium trap, ZS-9, is safe and efficient. *Kidney Int.* **2015**, *88*, 404–411. https://doi.org.10.1056/NEJMoa1810742

16. Packham, D.K.; Rasmussen, H.S.; Lavin, P.T.; El-Shahawy, M.A.; Roger, S.D.; Block, G.; Qunibi, W.; Pergola, P.; Singh B. Sodium zirconium cyclosilicate in hyperkalemia. *N. Engl. J. Med.* **2015**, *372*, 222–231. https://doi.org/10.1056/NEJMoa1411487
17. Tumlin, J.A.; Kosiborod, M.; Pergola, P.E.; Qunibi, W.E.; Packham, D.K.; Roger, S.D.; Lerma, E.V.; Fishbane, S.; Rasmussen, H.S.; Spinowitz. B.S. SA-PO1101: Long-term (52-Week) efficacy and safety of ZS-9 in the treatment of hyperkalemia: Interim results from a phase 3 open-label, multi-center, multi-dose maintenance study. *J. Am. Soc. Nephrol.* **2015**, *26*, B6. https://www.asn-online.org/education/kidneyweek/archives/KW15Abstracts.pdf
18. Fishbane, S.; Pergola, P.E.; Packham, D.K.; Roger, S.D.; Lerma, E.V.; Butler, J.; Von haehling, S.; Spinowitz, B.S.; Block, G.A.; *et al.* TH-PO1112: Efficacy and Safety of Sodium Zirconium Cyclosilicate for Hyperkalemia: 12-Month, Open-Label, Phase 3 Study. *J. Am. Soc. Nephrol.* **2017**, *28*, 390. https://www.asn-online.org/education/kidneyweek/archives/KW17Abstracts.pdf
19. Kosiborod, M.; Rasmussen, H.S.; Lavin, P.; Qunibi, W.Y.; Spinowitz, B.; Packham, D.; Roger, S.D.; Yang, A.; Lerma, E.; Singh, B. Effect of sodium zirconium cyclosilicate on potassium lowering for 28 days among outpatients with hyperkalemia: The HARMONIZE randomized clinical trial. *JAMA* **2014**, *312*, 2223–2233. https://doi.org/10.1001/jama.2014.15688
20. Anker, S.D.; Kosiborod, M.; Zannad, F.; Piña, I.L.; McCullough, P.A.; Filippatos, G.; van der Meer, P.; Ponikowski, P.; Rasmussen, H.S.; Lavin, P.T.; *et al.* Maintenance of serum potassium with sodium zirconium cyclosilicate (ZS-9) in heart failure patients: Results from a phase 3 randomized, double-blind, placebo-controlled trial. *Eur. J. Heart Fail.* **2015**, *17*, 1050–1056. https://doi.org/10.1002/ejhf.300
21. Lepage, L.; Dufour, A.C.; Doiron, J.; Handfield, K.; Desforges, K.; Bell, R.; Vallée, M.; Savoie, M.; Perreault, S.; Laurin, L.P.; *et al.* Randomized clinical trial of sodium polystyrene sulfonate for the treatment of mild hyperkalemia in CKD. *Clin. J. Am. Soc. Nephrol*. **2015**, *10*, 2136–2142. https://doi.org/10.2215/CJN.03640415
22. Pitt, B.; Bushinsky, D.A.; Kitzman, D.W.; Ruschitzka, F.; Metra, M.; Filippatos, G.; Rossignol, P.; Du Mond, C.; Garza, D.; Berman, L.; *et al.* Evaluation of an individualized dose titration regimen of patiromer to prevent hyperkalaemia in patients with heart failure and chronic kidney disease. *ESC Heart Fail.* **2018**, *5*, 257–266. https://doi.org/10.1002/ehf2.12265
23. Pitt, B.; Bushinsky, D.; Halfon, S.; Kitzman, D.; Lainscak, M.; Mathur, V.; Stasiv, Y.; Huang, I.-Z. 1085-44: A multicenter study of dose titration or the oral potassium binder RLY5016 to maintain normal serum potassium in patients with heart failure and chronic kidney disease treated with renin-angiotensin-aldosterone inhibitors and/or β-blockers. *J. Am. Coll. Cardiol.* **2011**, *57*, E301. https://doi.org/10.1016/S0735-1097(11)60301-X
24. Pitt, B.; Anker, S.D.; Bushinsky, D.A.; Kitzman, D.W.; Zannad, F.; Huang, I.Z. Evaluation of the efficacy and safety of RLY5016, a polymeric potassium binder, in a double-blind, placebo-controlled study in patients with chronic heart failure (the PEARL-HF) trial. *Eur. Heart J.* **2011**, *32*, 820–828. https://doi.org/10.1093/eurheartj/ehq502
25. Agarwal, R.; Rossignol, P.; Romero, A.; Garza, D.; Mayo, M.R.; Warren, S.; Ma, J.; White, W.B.; Williams, B. Patiromer versus placebo to enable spironolactone use in patients with resistant hypertension and chronic kidney disease (AMBER): a phase 2, randomised, double-blind, placebo-controlled trial. *Lancet* **2019**, *394*, 1540–1550. https://doi.org/10.1016/S0140-6736(19)32135-X
